# Modular nanotransporters: a versatile approach for enhancing nuclear delivery and cytotoxicity of Auger electron-emitting ^125^I

**DOI:** 10.1186/2191-219X-2-59

**Published:** 2012-10-29

**Authors:** Tatiana A Slastnikova, Eftychia Koumarianou, Andrey A Rosenkranz, Ganesan Vaidyanathan, Tatiana N Lupanova, Alexander S Sobolev, Michael R Zalutsky

**Affiliations:** 1 Laboratory of Molecular Genetics of Intracellular Transport, Institute of Gene Biology, Vavilov St. 34/5, Moscow, 119334, Russia; 2 Department of Biophysics, Faculty of Biology, Lomonosov Moscow State University, Leninskie Gory 1-12, Moscow, 119991, Russia; 3 Departments of Radiology, Duke University Medical Center, Durham, NC, 27710, USA; 4 Radiation Oncology, Duke University Medical Center, Durham, NC, 27710, USA; 5 Biomedical Engineering, Duke University Medical Center, Durham, NC, 27710, USA

**Keywords:** Modular nanotransporters, Auger radiotherapy, Iodine-125, Targeted delivery, EGFR

## Abstract

**Background:**

This study evaluates the potential utility of a modular nanotransporter (MNT) for enhancing the nuclear delivery and cytotoxicity of the Auger electron emitter ^125^I in cancer cells that overexpress the epidermal growth factor receptor (EGFR).

**Methods:**

MNTs are recombinant multifunctional polypeptides that we have developed for achieving selective delivery of short-range therapeutics into cancer cells. MNTs contain functional modules for receptor binding, internalization, endosomal escape and nuclear translocation, thereby facilitating the transport of drugs from the cell surface to the nucleus. The MNT described herein utilized EGF as the targeting ligand and was labeled with ^125^I using *N*-succinimidyl-4-guanidinomethyl-3-[^125^I]iodobenzoate (SGMIB). Membrane binding, intracellular and nuclear accumulation kinetics, and clonogenic survival assays were performed using the EGFR-expressing A431 epidermoid carcinoma and D247 MG glioma cell lines.

**Results:**

[^125^I]SGMIB-MNT bound to A431 and D247 MG cells with an affinity comparable to that of native EGF. More than 60% of internalized [^125^I]SGMIB-MNT radioactivity accumulated in the cell nuclei after a 1-h incubation. The cytotoxic effectiveness of [^125^I]SGMIB-MNT compared with ^125^I-labeled bovine serum albumin control was enhanced by a factor of 60 for D247 MG cells and more than 1,000-fold for A431 cells, which express higher levels of EGFR.

**Conclusions:**

MNT can be utilized to deliver ^125^I into the nuclei of cancer cells overexpressing EGFR, significantly enhancing cytotoxicity. Further evaluation of [^125^I]SGMIB-MNT as a targeted radiotherapeutic for EGFR-expressing cancer cells appears warranted.

## Background

Radionuclides emitting densely ionizing Auger electrons impart high cytotoxicity when they decay in close proximity to nuclear DNA due to the formation of double-strand breaks, resulting in severe DNA damage [1]. Because Auger electrons are characterized by short path lengths in the tissue and a very high linear energy transfer, their cytotoxic effects are limited to a sphere of a few nanometers in the immediate vicinity of the site of decay [2]. This property potentially makes them highly selective for killing targeted single cancer cells, provided that they can be specifically delivered to tumor cells, internalized, and transported to the cell nucleus [3].

For achieving selective delivery of short-range, highly cytotoxic therapeutics to their intended target cell populations, we have created engineered recombinant molecules - modular nanotransporters (MNT) - consisting of domains for accomplishing receptor binding and internalization as well as endosomal escape and nuclear translocation, thereby facilitating the delivery of drugs from the cell surface to the nucleus. We have recently documented the potential utility of MNT for delivering short range-of-action photosensitizers *in vitro*[4,5] and *in vivo*[6] with high cytotoxic effectiveness. Moreover, we have demonstrated proof of principle for using MNT as a platform for developing targeted short-range (α-particle) radiotherapeutic agents for cancer therapy [7]. Based on these encouraging results, we hypothesized that MNT might also be a useful vehicle for exploiting the high potency and subcellular range of Auger electrons for targeted radiotherapy, enabling specific delivery of Auger electron-emitting radionuclides to the highly radiosensitive cell nucleus of cancer cells.

A promising advantage of MNT is the interchangeable nature of the modules, offering the exciting prospect of generating an MNT or an MNT cocktail that possesses the best ligand, or mixture of ligands, and intracellular localizing signals that are tailored to the molecular profile of an individual patient’s tumor. The present study focuses on the development and the *in vitro* evaluation of a radiolabeled MNT possessing epidermal growth factor (EGF) as the ligand module, which ultimately might be suitable for targeting the many types of cancers overexpressing EGF receptors (EGFR) [8]. Iodine-125 was selected for these studies because it is the most well-studied Auger electron emitter ^125^I for targeted radiotherapy [9]. Although some of the properties of ^125^I (60-day half life, labeling chemistry) can present obstacles for eventual clinical translation, this radionuclide was selected to evaluate the feasibility of MNT mediated delivery of Auger electron emitters for several reasons. First, ^125^I is the most prototypic and well-studied Auger electron emitter for targeted radiotherapy [9]*.* And second, the fraction of total decay energy represented by Auger electron emission for ^125^I is higher than that for most alternative Auger electron emitters, including ^123^I, ^67^Ga, and ^111^In [1], making subcellular site of decay highly relevant. Binding, internalization, nuclear translocation and cytotoxicity were evaluated using two EGFR-expressing human cancer cell lines - A431 epidermoid carcinoma cells and D247 MG malignant glioma cells, which express varying levels of EGFR.

## Methods

### Cell lines

The human epidermoid carcinoma cell line A431 [10] was obtained from the ATCC (Manassas, VA, USA), and the human glioblastoma cell line D247 MG [11] was kindly provided by Dr. Darell Bigner, Duke University Medical Center. Previous studies in our laboratory indicated that the average number of EGFR per cell is 2.9 × 10^6^ and 2.4 × 10^5^ for A431 and D247 MG cells, respectively [7]. All tissue culture reagents were obtained from Gibco/Invitrogen (Carlsbad, CA, USA). The cells were cultured in zinc option medium supplemented with 10% fetal bovine serum and penicillin/streptomycin (100 U/mL) at 37**°**C in a 5% CO_2_ atmosphere.

### Modular nanotransporter

The MNT molecule used in these experiments was DTox-HMP-NLS-EGF (hereafter designated as MNT), which has a molecular weight of 76.3 kDa. DTox is the translocation domain of diphtheria toxin, serving as the endosomolytic module; HMP is an *Escherichia coli* hemoglobin-like protein, serving as the carrier module; the NLS is the optimized simian vacuolating virus 40 (SV40) large T-antigen nuclear localization sequence peptide; and EGF served as the ligand module [5]. The MNT was purified to >98% purity on Ni-NTA-agarose (QIAGEN, Hilden, Germany) according to the standard procedure furnished by the supplier. Each MNT module retained its intended function: high affinity interaction with EGFR and α/β-importin dimers, ensuring nuclear transport of NLS-containing protein, formation of holes in lipid bilayers at endosomal pH, and accumulation in the nuclei of A431 cells [5].

### Labeling MNT and EGF with ^125^I using *N*-succinimidyl-4-guanidinomethyl-3-[^125^I]iodobenzoate

No-carrier-added sodium ^125^I]iodide (2,200 Ci/mmol) was obtained from Perkin-Elmer Life and Analytical Sciences (Boston, MA, USA). A detailed method for the radiosynthesis of *N*-succinimidyl-4-guanidinomethyl-3-^125^I]iodobenzoate (SGMIB) and its use for labeling internalizing molecules have been described in a previous publication [12]. Briefly, a solution of the MNT (50 μL, 2 mg/mL), or human EGF (hEGF) (Sigma Chemical, St. Louis, MO, USA; 50 μL, 1 mg/mL) in 0.1 M borate buffer (pH 8.5) was added to ^125^I]SGMIB, and the mixture was incubated at room temperature for 20 min. The radiolabeled polypeptide conjugate was purified by gel filtration through a PD-10 column (GE Healthcare, Pittsburgh, PA, USA) that was eluted with phosphate-buffered saline (pH 7.5).

### Labeling with ^125^I using Iodogen

For radioligand assays, hEGF and bovine serum albumin (BSA) (Sigma Chemical, St. Louis, MO, USA) were labeled with ^125^I using Iodogen (Pierce, Rockford, IL, USA). The proteins and 1 to 3 mCi of sodium [^125^I]iodide in phosphate-buffered saline (pH 7.5) were incubated in glass vials coated with 10 μg of Iodogen for 15 min. Radioiodinated proteins were purified by passage through a PD-10 column as described above.

### Binding assays

The EGFR status of the two cell lines was confirmed by measuring ^125^I]iodoEGF binding, and the ability of MNT and ^125^I]SGMIB-MNT to bind specifically to EGFR was documented as previously described [7] prior to the initiation of any of the other experiments.

### Uptake and washout kinetics

The uptake and washout kinetics of ^125^I]SGMIB-MNT were measured on A431 and D247 MG cells in order to relate the cytotoxic effect to the number of ^125^I disintegrations. Cells were seeded in 24-well plates (5 × 10^4^ cells per well). Two days later, the cells were washed, and ^125^I]SGMIB-MNT (specific activity 12.8 mCi/mg) in media was added at a final concentration of 2.6 nM. The cells were incubated in triplicate for 0.5, 1, 2, 4, 8, 12, and 24 h in a humidified atmosphere at 37°C in 5% CO_2_. At each time point, an aliquot of media containing unbound radioactivity was collected, and the cells were washed with ice-cold Dulbecco's phosphate-buffered saline (DPBS) without calcium and magnesium. The cells were trypsinized and harvested for centrifugation. The membrane-bound radioactivity [13-15] released into the supernatant was collected, and the cells were resuspended in medium and centrifuged once more. The supernatant samples were collected along with the previous washes, while the pelleted cells were resuspended in medium. The internalized radioactivity from the resuspended cells, as well as the free radioactivity and the membrane-bound radioactivity samples, were counted for ^125^I activity using an automated gamma counter (Wallac Wizard 3”; Perkin-Elmer Life and Analytical Sciences, IL, USA). To determine washout kinetics, the cells were incubated with 0.3 nM of ^125^I]SGMIB-MNT (specific activity 6.0 mCi/mg) for 24 h at 37°C. After trypsinization and washing, the cells were resuspended in media and incubated for 0.5, 2, 4, 8, and 24 h at 37°C in 5% CO_2_. The washed out radioactivity was removed by centrifugation, followed by an additional washing of the cells with medium, and centrifuged again. After counting the ^125^I activity, the results were calculated as counts per minute per cell (CPM/cell) of internalized radioactivity as a function of time.

### Nuclear kinetics in A431 cells

The protocol for isolation of cell nuclei was based on a widely used method [16]. In brief, the cells were seeded in 6-well plates (5 × 10^5^ cells per well); 2 days later, the cells were washed, and ^125^I]SGMIB-MNT (specific activity 1.3 to 2.1 mCi/mg for these experiments) was added at a concentration (30 to 43 nM) selected based on the measured *K*_d_. Cells were incubated in triplicate for 1, 2, 4, 8, and 24 h at 37°C in 5% CO_2_. For the determination of nonspecific uptake, EGF in 100-fold excess also was added to the cells. At each time point, medium containing unbound radioactivity was collected, and the cells were washed three times with 1 ml of ice-cold DPBS without calcium and magnesium. The cells were trypsinized and harvested for centrifugation. The pelleted cells were resuspended in 0.5-mL ice-cold medium and centrifuged for a second time. The membrane-bound radioactivity released in these two supernatants was collected. The cells were swelled for 20 min on ice in 500 μL of ice-cold hypotonic buffer (25 mM Tris–HCl pH 7.5, 5 mM KCl, 0.5 mM dithiothreitol, 1 mM phenylmethanesulfonylfluoride, and 0.15 U/mL aprotinin), and 50 μL were removed for counting intracellular radioactivity. Following homogenization using a Dounce homogenizer on ice, the nuclei were pelleted by centrifugation at 600 × *g* for 10 min. Pelleted nuclei were washed three times with isotonic buffer (0.25 M sucrose, 6 mM MgCl_2_, 10 mM Tris–HCl, pH 7.4, 0.5% Triton X-100, 1 mM phenylmethanesulfonylfluoride, and 0.15 U/mL aprotinin) to remove any contamination from cytoplasmic membranes. The purity of the nuclear fraction was confirmed by careful microscopic evaluation and Western Blot analysis using antibodies to α-tubulin and histone deacetylase 1 (HDAC-1) as cytoplasmic and nuclear markers, respectively.

### Cytotoxicity study

Briefly, A431 or D247 MG cells were seeded in 24-well plates (5 × 10^4^ cells per well) and 2 days later were washed with media. A range of radioactive concentrations of [^125^I]SGMIB-MNT (0 to 101 μCi/mL), [^125^I]SGMIB-EGF (0 to 41 μCi/mL, only with A431 cells) or as a negative control, ^125^I-labeled bovine serum albumin ([^125^I]iodoBSA) (0 to 454 μCi/mL), were added, and the cells were incubated for 24 h in a humidified atmosphere at 37°C in 5% CO_2_. After the incubation, medium containing unbound radioactivity was removed, and the cells were washed twice with cold DPBS without calcium and magnesium. The cells were trypsinized and harvested prior to centrifugation. The supernatant representing the membrane-bound radioactivity was collected. The cells were resuspended in medium, and an aliquot was collected in order to assay the amount of internalized radioactivity using the automated gamma counter. The cells were counted, seeded for a colony-forming assay in 25-cm^2^ flasks (2,000 cells per flask), and maintained in a humidified atmosphere at 37°C in 5% CO_2_. After 6 to 7 days (A431 cells) or 11 to 14 days (D247 MG cells), the colonies were stained with crystal violet and were counted. The data were analyzed using GraphPad Prism Version 5.01 software (GraphPad, San Diego, CA, USA). Survival experiments were performed three separate times (A431 cells) or twice (D247 MG cells). Each experiment was done in triplicate.

### Calculation of decays accumulated inside the cell and nucleus

From the curves obtained for internalized CPM uptake and washout versus time (determined as described above in the uptake and washout kinetics section), the total number of ^125^I CPM delivered to the cells during the cytotoxicity experiment was calculated using the following equation:

(1) $$\text{CPM}=\overset{1,440\;\text{min}}{\underset{0\;\text{min}}{\int }}{\text{cpm}}_{1}\left(t\right)dt+\overset{+\infty }{\underset{1,440\;\text{min}}{\int }}{\text{cpm}}_{2}\left(t\right)dt\text{,}$$

where cpm_1_ (*t*) represents accumulation kinetics function of [^125^I]SGMIB-MNT and cpm_2_ (*t*) represents the washout kinetics function of [^125^I]SGMIB-MNT measured for each cell line. By correction for the detection efficiency of the gamma counter, the total number of disintegrations per cell was calculated.

The total number of decays accumulated per nucleus over the incubation was estimated using the following calculations and assumptions:

1) The area under the curve, expressed as the number of decays accumulated per nucleus over the incubation with predefined concentration of [^125^I]SGMIB-MNT, was calculated from the nuclear kinetics experiment data (0 to 24 h).

2) It was assumed that the percentage of decays that occurred per nucleus is constant over the range of [^125^I]SGMIB-MNT concentrations used in cytotoxicity studies.

Thus, decays occurring per nucleus were calculated for each concentration used in the cytotoxicity study according to the following equation:

(2) $$\text{decays per nucleus}=x_{0-24\;\text{h}}\cdot \overset{1,440\;\text{min}}{\underset{0\;\text{min}}{\int }}\frac{1}{a}{\text{cpm}}_{1}\left(t\right)dt+x_{24\;\text{h}}\cdot \overset{+\infty }{\underset{1,440\;\text{min}}{\int }}\frac{1}{a}\cdot {\text{cpm}}_{2}\left(t\right)dt\text{,}$$

where *a* is a factor which takes into account both the efficiency of the gamma counter (0.85 for ^125^I) and the abundance of its gamma photon used for measurement, $x_{0-24\;\text{h}}=\frac{\text{decays per nucleus}}{\text{total decays per cell}}$, accumulated over the 24-h incubation (this is obtained from the nuclear kinetics experiment, $x_{24\;\text{h}}=\frac{\text{decays per nucleus}}{\text{total decays per cell}}$ at the end of incubation (24 h), obtained from the nuclear kinetics experiment. $\overset{1,440\;\text{min}}{\underset{0\;\text{min}}{\int }}\frac{1}{a}\cdot {\text{cpm}}_{1}\left(t\right)dt$ and $\overset{+\infty }{\underset{1,440\;\text{min}}{\int }}\frac{1}{a}\cdot {\text{cpm}}_{2}\left(t\right)dt$were calculated as described above.

## Results and discussion

### Results

#### Labeling and quality control of [^125^I]SGMIB-MNT

**Figure 1:**
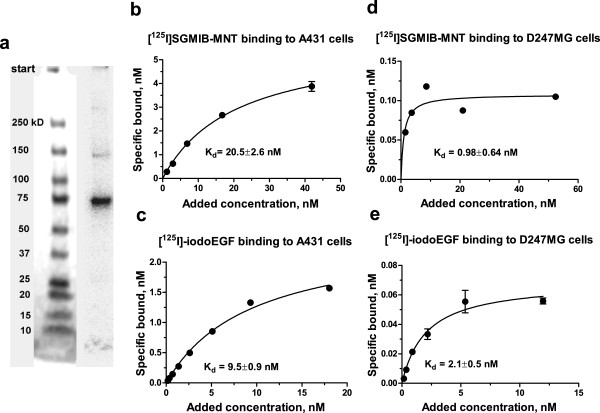
**Quality control of [**^**125**^**I]SGMIB-MNT**. (**a**) Phosphor image of the dried SDS-PAGE gel (Any kD™ Mini-PROTEAN® TGX™ Precast Gels, Bio-Rad, CA, USA) of [^125^I]SGMIB-MNT. The main band at about 75 kD standard band corresponds to full-sized [^125^I]SGMIB-MNT (57%), while the minor band at about 150 kD (12%) is consistent with an MNT dimer. (**b**) [^125^I]SGMIB-MNT and (**c**) [^125^I]iodoEGF binding to A431 cells. (**d**) [^125^I]SGMIB-MNT and (**e**) [^125^I]iodoEGF binding to D247 MG cells. Bars represent standard error of mean.

The yield for the MNT conjugation reaction was 60% to 80%, with >97% of the radioactivity protein associated, as determined by trichloroacetic acid precipitation. The specific activity of [^125^I]SGMIB-MNT varied from 1.3 to 12.8 mCi/mg. Gel electrophoresis of [^125^I]SGMIB-MNT and subsequent phosphor imaging of the dried gel showed one main band at about 75 kDa, which is consistent with the molecular weight of MNT (Figure 1a). The specific binding of [^125^I]SGMIB-MNT to EGFR-overexpressing A431 human epidermoid carcinoma and D247 MG human glioma cells (Figure 1b,d) as a function of concentration was similar to that observed for [^125^I]iodoEGF (Figure 1c,e). The binding affinity of [^125^I]SGMIB-MNT (*K*_d_ = 20.5 ± 2.6 nM), for EGFR measured on A431 cells was somewhat lower than that of [^125^I]iodoEGF (*K*_d_ = 9.5 ± 0.9 nM) but within the error of that obtained for unmodified MNT (*K*_d_ = 21.4 ± 2.6 nM; obtained from a competitive assay versus [^125^I]iodoEGF), suggesting that labeling did not compromise the MNT binding affinity. The binding affinity of [^125^I]SGMIB-MNT (*K*_d_ = 0.98 ± 0.64 nM) for EGFR measured on D247 MG cells was similar to that of [^125^I]iodoEGF (*K*_d_ = 2.1 ± 0.5 nM).

#### Binding/uptake and washout kinetics

**Figure 2:**
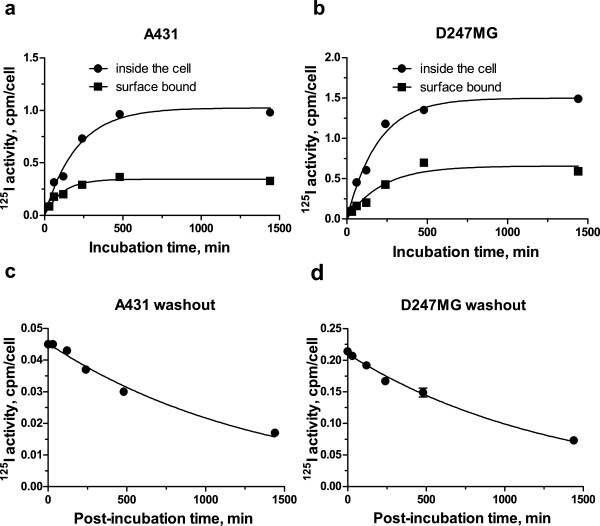
**[**^**125**^**I]SGMIB-MNT binding/uptake and washout kinetics**. Kinetics of binding and accumulation of [^125^I]SGMIB-MNT by (**a**) A431 human epidermoid carcinoma cells and (**b**) D247 MG human glioma cells. Total activity accumulated inside the cells (circles); total membrane-bound activity (squares). Washout kinetics of intracellular accumulated after 24 h of incubation [^125^I]SGMIB-MNT by (**c**) A431 human epidermoid carcinoma cells and (**d**) D247 MG human glioma cells. All data are expressed as activity in CPM per cell. For each data point, the experiment was done in triplicate with bars representing the standard error of mean.

The kinetics of membrane binding and internalization of [^125^I]SGMIB-MNT by A431 and D247 MG cells was investigated at selected time points up to 24 h. Intracellular and surface-bound radioactivity increased rapidly during the first 8 h of incubation, reaching near plateau values at 24 h (Figure 2a,b). Based on these results and taking into account the 60-day half-life of ^125^I, a 24-h incubation period was used for measuring washout kinetics and for performing the cytotoxicity assays. The initial washout kinetics of radioactivity from these EGFR-positive cell lines was rapid; however, approximately 30% of initially internalized radioactivity was retained inside the cells at 24 h (Figure 2c,d).

The exponential functions *y* = *y*_max_(1 − *e*^−*kx*^) and *y* = *y*_max_ − *e*^−*kx*^ were fit, respectively, to the experimental intracellular accumulation and washout kinetics data (CPM inside the cell versus time curves; Figure 2), with a good fit (*R*^2^ = 0.97) observed for both cell lines. The coefficients *y*_*max*_ and *k* were defined with the GraphPad Prism software from the kinetics data, for the fixed [^125^I]SGMIB-MNT concentration. The coefficient *k* was set by assuming that the shape of kinetics functions was independent of [^125^I]SGMIB-MNT concentration (*c*). We calculated *y*_max_(*c*) from the experimentally determined intracellular activity levels measured after a 24-h incubation at each concentration of [^125^I]SGMIB-MNT evaluated in the cytotoxicity experiment. Thus, we obtained cpm_1_ (*t*) and cpm_2_ (*t*) at each concentration.

#### Nuclear kinetics

**Figure 3:**
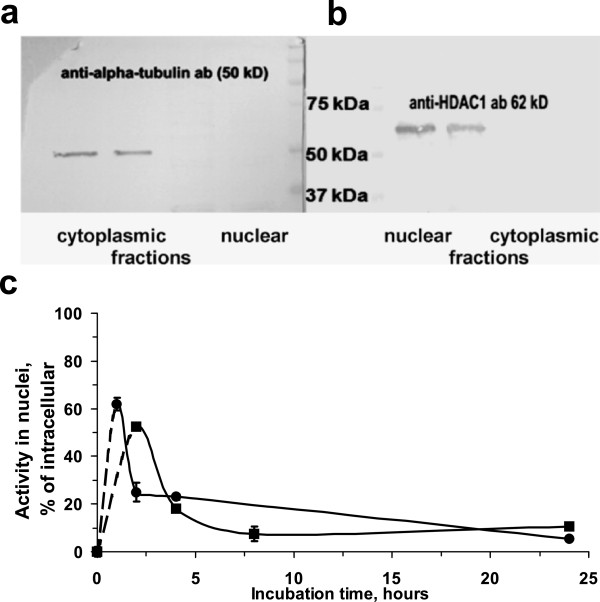
**[**^**125**^**I]SGMIB-MNT nuclear kinetics.** Western blot analysis for (**a**) α-tubulin (a cytoplasmic protein) and (**b**) histone deacytalase-1 (nuclear protein) in cytoplasmic fraction or nuclear fraction from A431 cells. (**c**) Nuclear kinetics of [^125^I]SGMIB-MNT in A431 cells (circles and squares refer to separate experiments). For each data point, the experiment was done in triplicate with bars representing the standard error of mean.

Because of the enhanced cytotoxicity of Auger electrons when localized in the cell nucleus, the dynamics of ^125^I localization in the cell nucleus after incubation of A431 cells with ^125^I]SGMIB-MNT was determined. The cell nuclei were isolated using a method previously reported by Lo et al. [16], which was successfully applied for the subcellular fractionation of A431 cells. Western blot analysis revealed negligible contamination of isolated nuclei with the abundant cytoplasmic protein, α-tubulin (Figure 3a), and low contamination of the cytoplasmic fraction with the soluble nuclear protein HDAC-1 (Figure 3b). The kinetics of nuclear accumulation of ^125^I]SGMIB-MNT was fast, with more than 60% of the total intracellular radioactivity in the nuclei after 1 h (Figure 3c). About 40% (from 0.27 CPM/nucleus at 1 h to 0.16 CPM/nucleus at 4 h) was redistributed from the cell nuclei over the next 3 h, gradually reducing to 0.07 CPM/nucleus at the end of the 24-h incubation. 

#### Cytotoxicity

**Figure 4:**
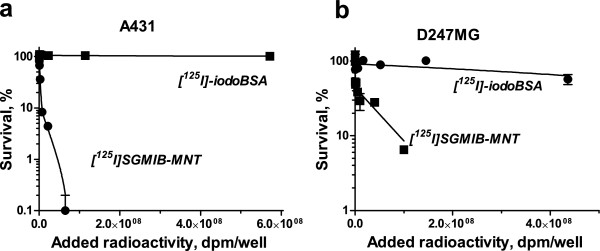
**Cytotoxicity of [**^**125**^**I]SGMIB-MNT**. Clonogenic survival of (**a**) A431 human epidermoid carcinoma cells and (**b**) D247 MG human glioma cells after exposure for 24 h to varying activity concentrations of [^125^I]SGMIB-MNT and [^125^I]iodoBSA. The experiments were performed three separate times (A431 cells) or twice (D247 MG cells) with survival curves of the representative experiment shown on the graph. For each data point, the experiment was done in triplicate with bars representing the standard error of mean.

**Figure 5:**
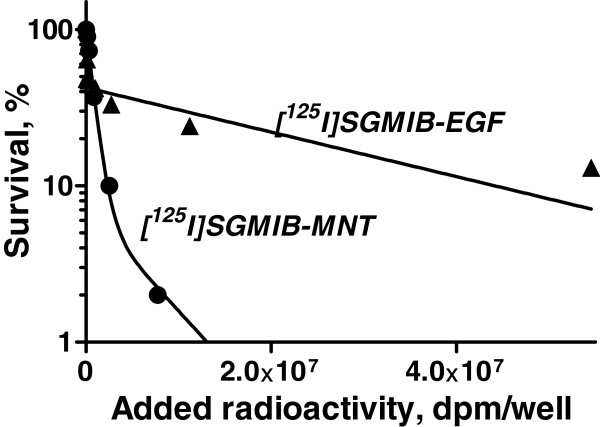
**Comparison of [**^**125**^**I]SGMIB-MNT and [**^**125**^**I]SGMIB-EGF cytotoxicity.** Clonogenic survival of A431 human epidermoid carcinoma cells after exposure for 24 h to varying activity concentrations of [^125^I]SGMIB-MNT (*A*_37_ = 9 × 10^5^ DPM per well; *A*_10_ = 2.4 × 10^6^ DPM per well) and [^125^I]SGMIB-EGF (*A*_37_ = 4 × 10^6^ DPM per well; *A*_10_ = 4.4 × 10^7^ DPM per well). For each data point, the experiment was done in triplicate with bars representing standard error of mean.

The clonogenic survival of EGFR-expressing A431 and D247 MG cells after a 24-h exposure to varying radioactivity concentrations of [^125^I]SGMIB-MNT and [^125^I]SGMIB-EGF and as a control for nonspecific cytotoxicity, [^125^I]iodoBSA, are presented in Figure 4 and Table 1 ([^125^I]SGMIB-MNT only). For [^125^I]SGMIB-MNT, the relationship between clonogenic survival and radioactivity concentration added to the medium was poorly fitted by a one-exponential model for both cell lines but were fitted effectively with two-exponential equations. The fitted curves were utilized to estimate the added radioactivity per well required to reduce survival to 37% (*A*_37_). The *A*_37_ values for [^125^I]SGMIB-MNT and [^125^I]iodoBSA were 2.7 × 10^6^ DPM (*N*_37_ ~ 3,000 decays per cell, or approximately 320 decays per nucleus) and approximately 1.5 × 10^10^ DPM (extrapolated), respectively, for A431 epidermoid carcinoma cells (Figure 4a, Table 1), and 1.2 × 10^7^ DPM (*N*_37_ ~ 3,800 decays per cell) and 7.6 × 10^8^ DPM, respectively, for D247 MG glioma cells (Figure 4b, Table 1). These results demonstrate that the cytotoxic effectiveness of [^125^I]SGMIB-MNT was 60 and more than 3,500 higher than that of nonspecific [^125^I]iodoBSA on the lower EGFR-expressing D247 MG and higher EGFR-expressing A431 cell lines, respectively. The cytotoxic effectiveness of [^125^I]SGMIB-MNT was 4.6 (when comparing *A*_37_) or 18.3 (when comparing more sensitive *A*_10_ values) times higher than that of [^125^I]SGMIB-EGF (Figure 5).

**Table 1:** **Cytotoxicity of [**^**125**^**I]SGMIB-MNT expressed as decays per cell or per nucleus**

**Cell line**	**A431**		**D247 MG**	
	**Mean**	**SE (*n*= 3)**	**Mean**	**SE (*n*= 2)**
*A*_37_ (decays per cell)	3,001	624	3,819	878
*A*_37_ (decays per nucleus)	317	66	N/D	N/D

N/D, not determined.

### Discussion

Due to the very short range of Auger electrons (0.15 to 22.5 nm in the tissue for ^125^I [17]), radionuclides that decay via Auger electron emission need to be delivered in close proximity to DNA within the nucleus to efficiently kill the target cells. The importance of localizing the site of decay in the cell nucleus can be illustrated by comparing the cellular *S* values (absorbed dose per unit cumulated activity) for ^125^I, considering a cell with cell and nuclear diameters of 18 and 10 μm, respectively, as an example [18]. In this case, shifting the site of decay from an extracellular site 1 μm from the cell surface, the cell membrane or the cytoplasm to the cell nucleus results in an enhancement of the radiation dose (gray per bequerel second) of 47, 39, and 21, respectively, highlighting the necessity of achieving a nuclear site of decay in order to maximize the therapeutic potential of Auger electron-emitting radionuclides. According to this formalism, the extent of enhancement calculated for a particular Auger electron emitter is dependent on the energetics and abundances of its Auger electron spectra as well as the geometry of the target cell. For some other Auger electron emitters that have been frequently investigated for targeted radiotherapy (^67^Ga, ^77^Br, ^111^In, and ^123^I), shifting the decay site from a cell surface receptor to the cell nucleus should increase the radiation dose received by the cell nucleus by at least a factor of 25 [18].

The most widely explored approach for delivering Auger electron emitters to the cell nucleus utilizes the thymidine analogue, 5-^125^I]iodo-2'-deoxyuridine (^125^IUdR), which can be incorporated into the DNA of cells in S-phase [19]. This strategy attempts to exploit the more rapid proliferation of malignant compared with normal cell populations. Although the limitations of IUdR are recognized, this agent provides an important point of reference because its effectiveness *in vitro* (in decays per cell/nucleus) reflects that achieved with ^125^I when nearly all its decays occur in DNA-incorporated form [19]. Thus, the *N*_37_ values expressed in decays per cell and decays per cell nucleus for ^125^IUdR are approximately the same. For Chinese hamster V79 lung fibroblasts, an *N*_37_ equal to 120 decays per cell nucleus has been reported [19] under the same experimental conditions utilized herein for determining the cytotoxicity of ^125^I]SGMIB-MNT on A431 cells, where *N*_37_ is estimated to be approximately 320 decays per nucleus.

In a previous study, we measured the cytotoxicity of ^125^IUdR for D247 MG glioma cells, one of the cell lines utilized in the current study [20]. The *A*_37_ determined after a 20-h incubation was 115 kBq/mL (3.1 μCi/mL). The cytotoxicity of ^125^I]SGMIB-MNT (9 μCi/mL) was somewhat lower than that reported for ^125^IUdR, both in terms of activity in the medium and *N*_37_, which is consistent with the fact that unlike the case of ^125^IUdR, ^125^I delivered by ^125^I]SGMIB-MNT would not be expected to be incorporated into the DNA.

However, it should be noted that cytotoxicity of Auger electron emitters, like other forms of radiation, can be influenced by the bystander effect, which is independent of the subcellular localization site of the radionuclide. For example, it was recently shown that this important, yet not fully understood, effect can be evoked by both nuclear (^123/125^IUDR) and mostly extra-nuclear (^123^I]MIBG) localized Auger electron emitters, modulating cytotoxicity of neighbor malignant cells [9,21,22].

Although ^125^IUdR has a considerable advantage from a cytotoxicity perspective, it also has significant limitations as an Auger-emitting targeted radiotherapeutic that have complicated its application in the clinical domain. These include an uptake mechanism limited to cells in S-phase, marginal tumor selectivity, extremely poor *in vivo* stability, and lack of suitability for use with radiometals such as ^111^In and ^67^Ga, which have half lives of about 3 days and thus could be more practical alternatives for clinical use than 60-day half life ^125^I. This has led a number of groups to evaluate proteins or peptides that attempt to increase the specificity of tumor association by targeting internalizing antigens or receptors expressed on the surface of cancer cells, generally in tandem with an NLS motif to promote nuclear translocation after receptor-/antigen-mediated internalization has occurred.

Pursuing this strategy, a number of groups have utilized internalizing antibodies [3,23,24] or ligands for internalizing receptors [25,26] preferably using residualizing labels to prolong the intracellular retention of radio activity, to deliver Auger electron emitters inside the cell. For these molecules, the *N*_37_, expressed generally in decays per cell, usually is much higher than that for ^125^IUdR. However, these constructs are often advantageous from the perspectives of specificity and *in vivo* stability. As an example, the *N*_37_ value for LL1 antibodies labeled with residualizing ^125^I-IMP-R2 was reported to be equal to 25,000 decays per cell [24]. The more favorable *N*_37_ value of approximately 3,000 decays per cell (varying from 1,760 to 3,733) for different specific activities used, obtained for MNT-mediated delivery of ^125^I]SGMIB, can be attributed to the fact that the MNT was designed not only to be internalized, but also to undergo nuclear targeting.

Being overexpressed on a great variety of cancer cells, EGFR is an attractive target for anticancer drug delivering systems. Anti-EGFR internalizing antibodies are being widely used for the targeted delivery of Auger electron emitters [9,23]. Michel et al. reported that a 48-h incubation with 40 μCi/mL of ^125^I-labeled anti-EGFR antibody was required to achieve 93% killing of EGFR-overexpressing A431 cells. In comparison, lower radioactivity concentration and shorter incubation time (5.6 to 11.4 μCi/mL and 24-h incubation) were needed to achieve 92% killing of A431 cells with ^125^I]SGMIB-MNT. We attribute these more favorable results to the presence of active nuclear-targeting signal on the MNT and the use of the residualizing labeling reagent, ^125^I]SGMIB. Moreover, our own data demonstrating significantly higher cytotoxicity of ^125^I]SGMIB-MNT compared to ^125^I]SGMIB-EGF (Figure 5) indicate that the active nuclear targeting itself enhances the effectiveness of EGFR-targeted drug delivery system.

In order to gain both tumor specificity and nuclear localization at the same time, several groups have utilized NLS-containing peptides [9] to route antibodies and peptides labeled with Auger electron-emitting radionuclides (e.g., ^111^In, ^99m^Tc) to the nucleus of cancer cells following their receptor-mediated internalization. Following this strategy, some investigators attached up to eight copies of the NLS sequence from SV-40 large T-antigen to various macromolecules, resulting in increased nuclear-associated radioactivity and reduced clonogenic survival [25,27].

However, prior to interaction with nuclear import machinery that is located in the cytoplasm, the NLS-bearing moiety needs to escape from the endosomes, where it can be trapped, after receptor binding induced internalization. Therefore, the endosomal escape module (DTox) was included in the MNT molecule to provide efficient ‘rescue’ of the construct, with the goal of increasing the probability of interaction with importins within the cytoplasm. The nuclear uptake of radioactivity, delivered by ^125^I]SGMIB-MNT, was about 60% of the internalized fraction after 1 h of incubation. This is very similar to that observed for an anti-CD33 antibody conjugated with up to eight NLS molecules, and incubated for the same 1-h period (66% of the internalized fraction in the nuclei) [27]; with one to four NLS molecules per macromolecule, approximately 25% to 30% of internalized radioactivity was in the nuclei [25,27]. Given that the MNT contains only one NLS, the observed higher nuclear-associated radioactivity could reflect the presence of the DTox module within the MNT.

The nuclear kinetics of ^111^ln-DTPA-hEGF that presumably follows the normal nuclear translocation of EGFR, previously reported by Reilly et al., was quite different from the results obtained for ^125^I]SGMIB-MNT [26]. The maximum radioactivity associated with the nuclei was 7% to 8% at 0.5 to 4 h for ^111^ln-DTPA-hEGF compared with 60% at 1 h for ^125^I]SGMIB-MNT presumably due to active nuclear targeting with NLS. However, contrary to ^111^ln-DTPA-hEGF (15% internalized at 24-h incubation), ^125^I]SGMIB-MNT nuclear kinetics showed a rapid decrease of nuclear-accumulated radioactivity (as a percentage of internalized radioactivity) with time (Figure 3c). Various naturally occurring processes or their combination could account for this behavior. At prolonged incubation times, radiation-induced stress and other toxicity effects of ^125^I]SGMIB-MNT could disrupt the normal function of NLS-mediated nuclear import [28]. Also, the MNT molecule, like many endogenous proteins, could be subjected to intranuclear proteasomal degradation [29]. The resultant small protein fragments and small molecular weight catabolites (with some fraction of them still labeled) could passively escape the nuclei through the nuclear pores.

As the nuclear retention of radioactivity reaches its maximum values during the first few hours of incubation, this specific MNT molecule probably would be better suited for targeted delivery of shorter half-life Auger electron emitters, for example ^123^I (13.2 h half-life compared to 60 days for ^125^I). In this case, a higher percentage of total decays would occur during the first few hours, when the portion of activity in the nuclei is maximal, thereby leading to more efficient cell kill. On the other hand, for the delivery of long half-life radionuclides like ^125^I, other possible approaches need to be considered to fully exploit the potential of [^125^I]SGMIB-MNT. These may include a partial modification of the MNT molecule in order to make it less liable to proteasomal degradation or trapping the radiolabel in the cell nuclei by chemical modification of the labeling site.

Previously, we evaluated the cytotoxicity of this MNT labeled with α-particle-emitting radiohalogen ^211^At using the SGMIB analogue - SAGMB. ^211^At]SAGMB-MNT resulted in 10 to 20 times more cytotoxicity than ^211^At]astatide for the same cell lines used in the present study, with *A*_37_ values between 3.8 and 19.7 kBq/mL (0.1 and 0.5 μCi/mL) depending on the cell line [7]. On the other hand, consistent with the much shorter range of Auger electrons compared with α-particles, the cytotoxicity of ^125^I]SGMIB-MNT was much more specific with a 60- to >3,500-fold higher cytotoxicity than that seen for nonspecific ^125^I]iodoBSA, compared with a specificity factor of about 7 for the α-emitter. This is consistent with the mostly subcellular range of ^125^I emissions, narrowing the site of action almost to the cell nuclei [9].

Given that MNT incorporates two bacterial proteins (DTox and HMP), the clinical translation of this modular agent might be questionable. Although the studies were preliminary in nature, we have shown that this MNT as well as another MNT incorporating the same DTox-HMP-NLS modules but with α-melanocyte-stimulating hormone as the ligand module were not toxic to mice when administered either as a single injection at the maximum achievable dose or when administered in multiple dose regimens [6]. In addition, the latter MNT elicited only a minor delayed hypersensitivity response in mice, suggesting a low degree of immunogenicity for this polypeptide.

## Conclusions

In the present study, we have demonstrated that MNT shows promise as a new platform for targeted radiotherapy using Auger electron-emitting radionuclides. The EGFR-targeted MNT, after labeling with the residualizing [^125^I]SGMIB prosthetic group, bound to EGFR-expressing tumor cells with an affinity comparable to that of native EGF. The [^125^I]SGMIB-MNT conjugate was rapidly internalized, with more than 60% of internalized [^125^I]SGMIB-MNT radioactivity accumulating in the cell nuclei after a 1-h incubation. Consistent with this, the cytotoxic effectiveness of [^125^I]SGMIB-MNT compared to non-internalized ^125^I-labeled bovine serum albumin control was enhanced by a factor of 60 to more than 3,700-fold, depending on the EGFR expression level of the cell line. Moreover, the cytotoxicity of [^125^I]SGMIB-MNT was considerably higher than that of the [^125^I]SGMIB-EGF, confirming potential advantages of the MNT platform for Auger electron-targeted radiotherapy. Taken together, these results suggest that MNT warrants further evaluation for this purpose, ideally, in tandem with Auger electron emitters such as ^111^In and ^67^Ga or ^123^I with half lives better matched to the intracellular kinetics observed herein and more appropriate for clinical translation.

## Abbreviations

BSA: Bovine serum albumin; CPM: Counts per minute; DPBS: Dulbecco's phosphate-buffered saline; DPM: Decays per minute; DTPA: Diethylenetriaminepentaacetic acid; DTox: Translocation domain of diphtheria toxin; EGF: Epidermal growth factor; EGFR: Epidermal growth factor receptor; HDAC-1: Histone deacetylase 1; HMP: *E*. *coli* hemoglobin-like protein; IUdR: 5-[^125^I]iodo-2'-deoxyuridine; MNT: Modular nanotransporter; NLS: Nuclear localization sequence; SGMIB: *N*-succinimidyl-4-guanidinomethyl-3-[^125^I]iodobenzoate.

## Competing interests

The authors declare that they have no competing interests.

## Authors’ contributions

TAS designed the study, performed the experiments, analyzed and interpreted the data, and drafted and wrote the manuscript. EK designed the study, performed the experiments, analyzed and interpreted the data, and wrote the manuscript. AAR, GV, ASS, and MRZ designed the study, analyzed and interpreted the data, and wrote the manuscript. TNL performed the experiments. All authors read and approved the final manuscript.
